# Sentiment Dynamics Among Informal Caregivers in Web-Based Alzheimer Communities: Systematic Analysis of Emotional Support and Interaction Patterns

**DOI:** 10.2196/60050

**Published:** 2024-12-04

**Authors:** Congning Ni, Qingyuan Song, Qingxia Chen, Lijun Song, Patricia Commiskey, Lauren Stratton, Bradley Malin, Zhijun Yin

**Affiliations:** 1 Department of Computer Science Vanderbilt University Nashville, TN United States; 2 Department of Biomedical Informatics Vanderbilt University Medical Center Nashville, TN United States; 3 Department of Biostatistics Vanderbilt University Medical Center Nashville, TN United States; 4 Department of Sociology Vanderbilt University Nashville, TN United States; 5 Department of Neurology Vanderbilt University Medical Center NASHVILLE, TN United States; 6 Psychosocial Research and Program Evaluation Alzheimer’s Association Chicago, IL United States; 7 Center for Genetic Privacy & Identity in Community Settings Vanderbilt University Medical Center Nashville, TN United States

**Keywords:** informal caregivers, Alzheimer disease, dementias, web-based community, sentiment analysis, topic modeling, caregiving, carers, family care, support group, peer support, gerontology, geriatrics, aging, attitudes, opinion, perceptions, perspectives, sentiment, cognitive, web-based communities, Linguistic Inquiry and Word Count, machine learning, Valence Aware Dictionary for Sentiment Reasoning

## Abstract

**Background:**

Alzheimer disease and related dementias (ADRD) are a growing global health challenge. ADRD place significant physical, emotional, and financial burdens on informal caregivers and negatively affects their well-being. Web-based social media platforms have emerged as valuable sources of peer support for these caregivers. However, there has been limited investigation into how web-based peer support might influence their mental well-being.

**Objective:**

This study aims to examine the dynamics of sentiment scores, a major indicator of mental well-being, among informal ADRD caregivers, specifically how their sentiment changes as they participate in caregiving experience discussions within 2 ADRD web-based communities.

**Methods:**

We collected data from 2 large web-based ADRD caregiving communities, ALZConnected (from November 2011 to August 2022) and TalkingPoint (from March 2003 to November 2022). Using the Valence Aware Dictionary for Sentiment Reasoning and Linguistic Inquiry and Word Count, we calculated sentiment scores for each post and evaluated how the initial sentiment score of a topic initiator evolves within a discussion thread. Structured topic modeling and regression analysis were used to identify the primary topics consistently associated with sentiment changes within these threads. We investigated longitudinal sentiment trends to identify patterns of sentimental stability or enhancement due to prolonged engagement in web-based communities by plotting linear interpolation lines of the sentiment values of each individual user.

**Results:**

The ALZConnected dataset comprised 532,992 posts, consisting of 57,641 topic threads and 475,351 comments. The TalkingPoint dataset was composed of 846,344 posts, consisting of 81,068 topic threads and 765,276 comments. Our research revealed that topic initiators experienced a notable increase in sentiment as they engaged in subsequent discussions within their threads, with a significant uptick in positivity in the short term. This phenomenon is part of a broader trend of steadily rising positive sentiment among ADRD caregivers. Using structured topic modeling, we cataloged a diverse range of topics that included both emotional aspects, such as family emotions, and practical concerns, such as diagnosis and treatment and everyday care practices. We observed that sentiment scores were positively aligned with discussions about family and daily routines life (coefficient=3.53; *P*<.001), while topics related to illness (coefficient=–1.37; *P*<.001) and caregiving facilities (coefficient=–1.98; *P*<.001) tended to correlate with lower sentiment scores. This evidence highlights the significant impact that both the time of participation and the posting content have on the sentiment changes of caregivers.

**Conclusions:**

This study identifies sentiment changes among informal ADRD caregivers through their interactions in 2 extensive web-based communities. These findings emphasize the importance of early emotional support within a topic thread and demonstrate a predominantly positive sentiment in these communities over time. These further highlight the value of web-based peer support and its potential to enhance the emotional well-being of informal ADRD caregivers.

## Introduction

Alzheimer disease (AD) is the most common cause of dementia, a clinical syndrome that severely impairs a person’s memory, language, and judgment and planning abilities [1]. AD and related dementias (ADRD) are increasingly prevalent health issues all over the world [2]. The responsibility of caring for people living with ADRD falls primarily on the unpaid informal caregivers, typically the family members and friends of the people living with ADRD [3,4]. These informal caregivers face a wide range of physical, emotional, and financial challenges that can cause significant stress and negatively affect their health and well-being [5]. Notably, many informal ADRD caregivers claim to have emotional exhaustion that manifests in caregivers as a result of prolonged emotional stress and the constant demands of caregiving. [6].

Understanding the emotional challenges informal ADRD caregivers face and offering appropriate support to enhance their well-being is of vital importance. Prior investigations into these issues have primarily relied upon traditional offline strategies, such as surveys [7,8] and interventions [9]. Additionally, the primary support for ADRD caregivers mainly comes from local, offline resources like charities and community support groups, with efforts like those of Meyers et al [10], who are committed to advancing ADRD research through collaborating with funded researchers and communities. The study by Robinson et al [11] uses existing cross-sectional survey data from the National Institute of Nursing Research–funded National Caregiver Training Project to examine differences between users and nonusers of community services among caregivers of persons with dementia. While these methodologies play an important role in studying and assisting informal ADRD caregivers’ needs, they come with inherent challenges. Caregivers may face hurdles in accessing offline support due to geographic constraints [12], resource limitations [13], and individual preferences. As for researchers, offline studies demand financial and human resources and can introduce potential geographical and demographic biases in the collected data.

Recognizing the limitations of offline support, social media platforms have emerged as a valuable, convenient resource for caregivers to gain informational and emotional support that may not be easily obtained in traditional offline face-to-face interactions [14,15]. A recent survey indicated that web-based communities could provide informal caregivers with a sense of understanding, empowerment, support, and belongingness, thus reducing social isolation and improving the emotional well-being of these caregivers [16]. Despite the potential benefits of web-based support, there is a lack of research examining whether informal ADRD caregivers receive positive emotional feedback when discussing their caregiving experience or challenges in web-based communities. This is important because an improved sentiment change observed from posts on web-based platforms may indicate a positive impact of web-based peer support on a caregiver’s emotional well-being [17].

In this study, we investigate the changes in the sentiment exhibited by informal ADRD caregivers through their published posts on web-based platforms in 2 large web-based communities, ALZConnected and TalkingPoint. ALZConnected is a web-based community powered by the Alzheimer’s Association for any person affected by ADRD in North America, while TalkingPoint is a web-based ADRD community organized by the UK Alzheimer’s Society. The selection of these platforms was based on their substantial user base, which provides a rich dataset for analysis, and their focus on ADRD, ensuring that the study’s insights are directly applicable to this group. Web-based peer support has been shown to offer a wide range of benefits, including informational and emotional support [18,19], which should also be valuable for caregivers facing the complex challenges of ADRD. Therefore, we hypothesize that the sentiment of informal ADRD caregivers revealed in their published posts will be improved after interacting with other caregivers in web-based communities. Specifically, we investigated the following 3 research questions (RQs) to test this hypothesis.

- RQ1: How did the sentiment of the topic initiator change within a topic thread?
- RQ2: What topics in initial posts were associated with sentiment change?
- RQ3: How did the sentiment of a web-based caregiver change over time within the community?

To investigate these questions, we apply sentiment analysis and statistical methods to determine whether engaging in web-based communities can provide emotional benefits to informal ADRD caregivers.

## Methods

For context, there are several key terms that we rely upon in this paper. Web-based communities generally structure discussions into disparate topic threads. Each thread is defined as an initial post followed by several subsequent comments, where these comments contribute to the ongoing discussion initiated by the original post. We refer to the user who initiates a topic thread as the “topic initiator” of the topic thread. The comments published by topic initiators within their own topic threads are called “self-comments.”

### Data Collection and Preprocessing

We collected data from two large, representative web-based communities that create a unique environment for ADRD caregiving discussions: (1) ALZConnected and (2) TalkingPoint. ALZConnected was established by the Alzheimer’s Association [20] as the first and the largest web-based community for any person affected by ADRD in North America. TalkingPoint, on the other hand, is a web-based community organized by the Alzheimer’s Society [21] in the United Kingdom for people living with ADRD or their caregivers to share information, advice, and support with one another.

We focused our analysis on a specific subset of forums that are dedicated to ADRD caregivers who share caregiving experiences, seek assistance, and engage in caregiving discussions. To ensure relevance and coherence, we conducted a preliminary selection process, manually reviewing the top 20 most viewed or commented posts within each relevant forum to assess their alignment with caregiving topics. This selection process was designed to retain the vast majority of relevant posts while excluding those that were not relevant to our study, such as posts from individuals with ADRD themselves or from forum administrators. In TalkingPoint, we focused on users with a label of a registered user or new member*.* In ALZConnected, we focused on users who self-identified as ADRD caregivers. The selection criteria varied between forums due to differences in their search functionalities and user engagement metrics.

We gathered all publicly accessible data from these 2 communities using a web crawler built with the *BeautifulSoup* package of Python (version 4.11; Python Software Foundation). We removed punctuation, special characters, and emojis, and converted the text to lowercase.

### Ethical Considerations

Our study received an exemption from human participants research by the institutional review board at Vanderbilt University Medical Center (IRB 221732). Informed consent was waived due to the study’s exempt status. To ensure participant privacy and confidentiality, all quoted texts have been paraphrased to prevent user identification. No compensation was provided to participants, as the research involved minimal risk and did not require direct interaction.

### Sentiment Evaluation

To mitigate measurement bias that can result from applying off-the-shelf models to our dataset, we applied 2 popular sentiment analysis tools, specifically, Valence Aware Dictionary for Sentiment Reasoning (VADER) [22] and Linguistic Inquiry and Word Count (LIWC) [23], to calculate the sentiment scores of web-based communications to quantify the overall sentiment expressed. These tools were chosen for their ability to consistently analyze large volumes of text data, making them suitable for our study. While they do not have traditional performance metrics like accuracy or *F*_1_-score, they have been validated in numerous studies for their reliability in sentiment analysis.

VADER is a module of The Natural Language Toolkit [24] that provides sentiment ratings based on the words used. It operates as a rule-based sentiment analyzer, where terms are categorized as positive or negative based on their semantic orientation. In this study, we selected the VADER compound score, calculated by summing the valence scores of each word in the lexicon and then normalizing it to a range between –1=most extreme negative and +1=most extreme positive as the sentiment evaluation score.

LIWC calculates the percentage of words in each linguistic category by mapping the words of a given text into a predefined word list of that category [25]. This tool has been widely adopted in social media content–based research [26]. In this study, we focused on the tone category in LIWC, which summarizes the 2 dimensions of positive and negative emotions into a single variable. The LIWC tone score ranged from 0% to 100%, with higher scores indicating a more positive emotional tone. The delineation occurred at 50%, where scores above (below) indicated a positive (negative) tone. In this study, we standardized the LIWC tone score to a range of –1 to 1 to align it with the VADER score range.

The sentiment score, as calculated by VADER and LIWC, reflects the emotional tone inferred from the text and serves as a proxy for emotional support in our study. For example, the post “Hi kids! I want to take a moment to thank all the veterans and their families on this forum...” received a predominantly positive sentiment (VADER 0.94, LIWC tone 0.98), while the post “My mother has lived with my husband and me for a year and I have always felt frustrated and resentful towards her...” was evaluated as predominantly negative (VADER –0.96, LIWC tone –0.84). “Emotional support” is defined here as the presence of supportive feedback inferred from positive shifts in sentiment scores following responses to a user’s posts, which will guide our analysis of the changes in sentiment throughout the subsequent research questions.

### RQ1: Sentiment Changes of Topic Initiators

To measure a topic initiator’s sentiment score changes within a topic thread, we focused on the topic initiators who published at least *M* (*M*>0) self-comments with a topic thread. This threshold ensures that the topic initiator contributes sufficient conversational involvement. In this study, *M* was set to a value that ensured at least 95% of the users posted at most *M* self-comments.

For each topic thread with at least *M* self-comments, we define an array, 
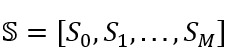
, to represent the sentiment scores of the initial post and the following *M* self-comments in chronological order. As such, *S*_0_ is the sentiment score of the initial post, while the *S*_i_ where i ∈ {1,...,*M*} is the sentiment score of the *i*th self-comment. We defined sentiment change, *S*_Δ_, as the difference between the average sentiment score of *m* self-comments and the initial post’s sentiment score: 
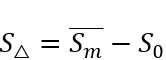
, where 
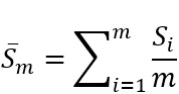
, and *m* ≤ *M*. We analyzed the sentiment score changes and generated distributions of these changes with a 95% CI.

To address potential bias from highly active users who contributed a large number of topic threads, we repeated the comparison by randomly selecting a group of P ∈ {5%,10%,25%,50%} of the total number of topic threads in each community. We conducted a pairwise 2-tailed *t* test to evaluate the difference between 
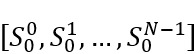
 and 
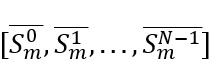
, where *N* represents the number of selected topic threads in each comparison. We examined the difference at the significance level of α=.05/4 with Bonferroni correction. This adjustment ensures that the error rate remains at the conventional 5% level across all 4 testing groups.

### RQ2: Association Between Sentiment Changes and Initial Post Topics

We used the structural topic model (STM) [27] to infer the topics that were communicated in the initial posts, with the subsequent goal of exploring their correlation with sentiment changes. STM is an advanced modeling technique that allows for the incorporation of document-level metadata to inform the discovery of topics within textual data. This unsupervised machine learning approach is particularly adept at handling large, unstructured datasets by identifying latent thematic structures without the need for preassigned labels. This capability makes it an excellent tool for exploring the vast and varied content found in web-based caregiver discussions, where topics may not be clearly defined in advance. Next, we applied ordinary least squares regression, as implemented in the Python package *statmodels* (version 0.14.0), to investigate what kinds of topics in the initial posts are associated with sentiment change.

Before applying topic modeling on the initial posts, we removed stop words and special symbols and discarded words that occurred less than 10 times in the dataset. Since STM is an unsupervised machine learning strategy, we rely on 2 metrics—exclusivity and semantic coherence—to determine the appropriate number of generated topics. Exclusivity refers to the uniqueness of the most frequent words in a topic, while semantic coherence [28] quantifies the co-occurrence of words in a topic in a general context or all the posts. We assess STM for topic numbers ranging from 5 to 30 and select the optimal number *K* of topics for further analysis [14].

Subsequently, we rank the topics by their prevalence across all documents, a process that involves examining the expected proportion of words in each document attributed to each topic. We calculate the expected topic proportions (ETP) using the *estimateEffect* function in the *STM* package. With this distribution, we conducted a regression analysis where the topic proportions served as independent variables, and the changes in sentiment scores, which were calculated by VADER compound scores or LIWC tone changes, served as the dependent variable. In this regression, we only considered the topics that held an ETP greater than the mean ETP of *K* topics. For instance, in a model with 20 topics, we would expect an ETP of 5% per topic (1%/20 or 100%/20) on average and only include topics that exceeded 5% in the regression analysis for each initial post.

### RQ3: Temporal Changes of Caregiver Sentiment

We defined the active time of a web-based caregiver in the community, up to the point of writing a specific post, as the duration from their account registration to the posting date of that particular post. We analyzed sentiment changes over various fixed time intervals, as 1 week, 2 weeks, 1 month, 3 months, 6 months, 1 year, 3 years, 5 years, and 10 years. For each time interval, we selected posts from active users who had contributed within the designated time frame (0 to the specific interval) and continued to post at least once after that period. For example, to assess sentiment changes for active users within 1 year, we only consider users who have published at least 2 posts (either initial posts or comments) within a year and still have at least 1 post beyond the 1-year period. As such, we ensure that every user included in a time-period analysis is still active in contributing posts. We focused on time intervals where at least half of the users remained active.

To quantify the change in sentiment for active users over time, we plotted linear interpolation lines with 95% CIs to analyze trends in the sentiment values of each individual user. We calculated the Spearman coefficient of correlation [29] to analyze similarities in trends across communities, as well as to validate results across different sentiment analysis tools.

## Results

### Basic Statistics

We collected data from ALZConnected from November 14, 2011, to August 6, 2022, and TalkingPoint from March 31, 2003, to November 3, 2022. Table 1 provides basic statistics for both datasets. The different time periods for each dataset reflect the respective forum’s establishment dates and the availability of their archival data, with no intercommunity comparison being made.

**Table 1:** Summary statistics for the datasets in this study.

Community	Total posts, n	Topic threads, n	Comments, n	Authors, n	Topic initiators, n	Commentors, n	Time period
ALZConnected	532,992	57,641	475,351	18,569	12,590	14,964	From November 14, 2011, to August 6, 2022
TalkingPoint	846,344	81,068	765,276	34,551	27,907	26,651	From March 31, 2003, to November 3, 2022

The ALZConnected dataset covers 532,992 posts, consisting of 57,641 topic threads and 475,351 comments. It involves 18,569 unique users, 12,590 (68%) of which are topic initiators and 14,964 (80.1%) are commenters, indicating that a nontrivial proportion of users engage in both creating and discussing content. The TalkingPoint dataset covers 846,344 posts, consisting of 81,068 topic threads and 765,276 comments. It involves 34,551 unique users, 27,907 (81%) of which are topic initiators and 26,651 (77.1%) are commenters.

**Figure 1:**
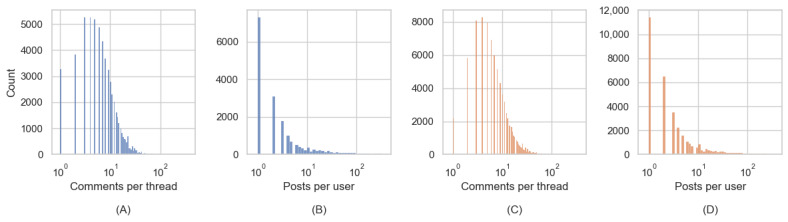
(A,C) The distribution of the number of comments per topic thread and (B,D) the number of posts per user in the ALZConnected and TalkingPoint web-based communities. The plots show the log-scaled x-axis for ease of viewing.

Both datasets exhibit a long-tailed distribution with respect to the number of comments per topic thread (Figures 1A and 1C). For example, most topic threads contain around 10 comments while a few inspire extensive dialogue. Similarly, the distribution of posts per user (Figures 1B and 1D) indicates that, although many users occasionally participate, a small subset of highly active users contribute the majority of the content. These phenomena hold true in both ALZConnected and TalkingPoint. The consistency in posting and user activity patterns across both communities highlights common behaviors in user engagement within social media caregiving forums.

### RQ1: Sentiment Change of Topic Initiator

After removing threads lacking self-comments, 30,739 (53.3%) out of 57,641 topic threads from ALZConnected and 53,995 (67%) out of 81,068 topic threads from TalkingPoint remained, comprising 79,869 and 181,049 self-comments, respectively. In ALZConnected, 95% of the topic initiators have fewer than 6 self-comments, while in TalkingPoint, 95% of the topic initiators have fewer than 8 self-comments. To minimize the influence of highly active users, we focused on measuring sentiment changes for the first *M*=10 self-comments, encompassing 30,209 (98.3%) out of 30,739 and 52,370 (97%) out of 53,995 topic threads, respectively, in ALZConnected and TalkingPoint.

**Figure 2:**
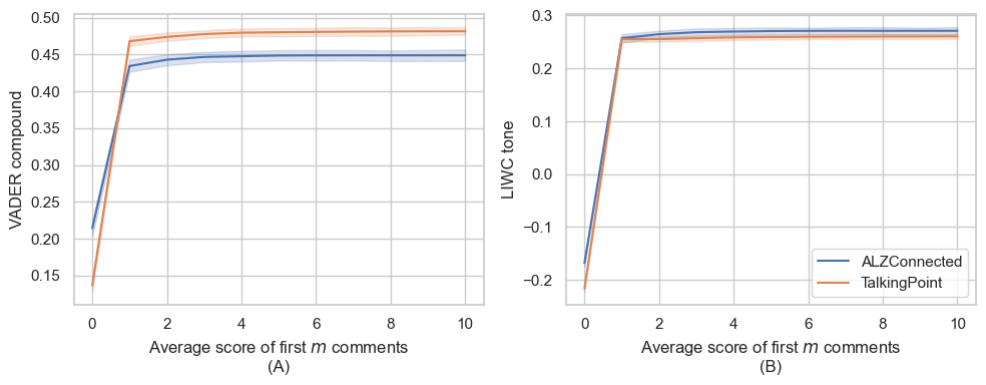
Sentiment changes as a function of the number of self-comments. The x-axis indicates the count of self-comments. For example, m=2 represents the average sentiment of the first 2 self-comments under each author’s thread. LIWC: Linguistic Inquiry and Word Count; VADER: Valence Aware Dictionary for Sentiment Reasoning.

Figure 2 shows how the sentiment score changes within a 95% CI, in terms of VADER compound (Figure 2A) and LIWC tone (Figure 2B), throughout the count of self-comments *m*. The *m*=0 on the x-axis corresponds to the initial sentiment score *S*_0_ while *m*>0 corresponds to the average score of the first *m* self-comments 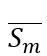. For example, a post in ALZConnected stated, “I am overwhelmed with sadness. This entire week, my husband’s condition has worsened, leaving him fixated on repetitive thoughts that I cannot divert…[rephrased]” with initial sentiment scores (VADER –0.85, LIWC –0.88). After receiving comments from 3 users, the caregiver’s follow-up post showed, “Thank you for your response...My husband, diagnosed with early onset Alzheimer’s 5 years ago, isn’t physically ill, yet we face immense challenges. [rephrased]” with sentiment scores improving to (VADER 0.54, LIWC –0.48). Thus, it is evident that initial engagement in web-based community discussions is associated with a notable increase in sentiment scores, suggesting prompt emotional support for topic initiators (RQ1). Both VADER and LIWC indicate a substantial increase in the sentiment score as the number of self-comments *m* grows from 0 to 1. This is followed by a slow, gradual increase as the number of self-comments further increases. This suggests that web-based community interactions effectively provide prompt emotional support to topic initiators. However, these positive changes in sentiment do not escalate rapidly with the frequency of topic initiators’ activities within the web-based communities.

VADER and LIWC show different patterns of changes in sentiment scores. Notably, the VADER compound score indicates a higher initial sentiment in ALZConnected compared to TalkingPoint. However, when the number of self-comments exceeds 1, the sentiment value in TalkingPoint surpasses that of ALZConnected. Conversely, the LIWC tone score shows an opposite trend, though the differences between the 2 communities are less distinct. This dissimilarity might arise from variations in the training corpora used by VADER and LIWC.

We investigated whether the initial sentiment significantly differed from the average sentiment across the first 10 self-comments. The results indicated statistically significant differences between the 2 sentiment values in both web-based communities for all test groups, with *P* values approaching 0, significantly lower than the significance level of α=.0125 (Bonferroni-corrected from α=.05/4). These findings emphasize that web-based interactions have a prompt and noticeable impact on the sentiment of caregivers in web-based communities.

### RQ2: Sentiment Changes Correlates with Initial Post Topics

**Figure 3:**
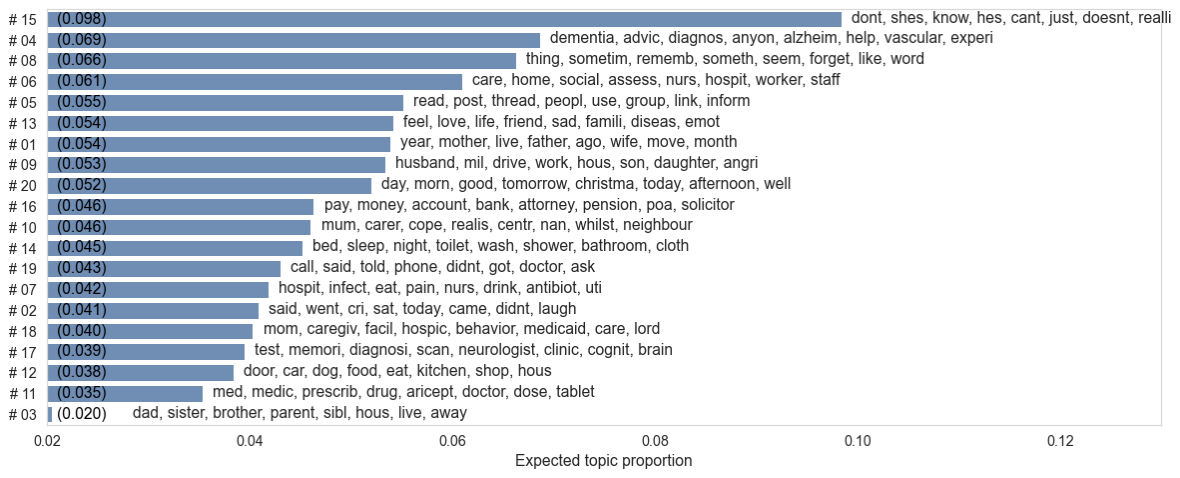
Topics generated by structural topic modeling, sorted in decreasing order of expected topic proportions. The proportion of each topic is shown to the right of each bar, while the top 8 most representative words in the topic are shown to the right.

In determining the ideal number of topics for our STM, we evaluated metrics of exclusivity and semantic coherence across a spectrum ranging from 5 to 30 topics. The analysis indicated that a set of 20 topics achieved an optimal balance between word distinctiveness and thematic relevance. Thus, we retrained STM on this number of topics. Figure 3 visualizes this topic modeling, showing the most representative words for each topic and indicating the relative topic proportions, which quantitatively reflect the prevalence of each topic across all analyzed documents.

From Figure 3, it was evident that several topics were related to sentiment. For instance, topic #13, which is about “family members feelings,” is characterized by frequent words like “feel,” “love,” and “sad.” The ETP of this topic is 5.4%, which stands out as notably high when considering an even distribution across 20 topics would average 5% per topic. This suggests that discussions related to family members’ feelings are more prevalent in the dataset than what would be expected by chance. The following posts are representative examples of this topic: “Sadly, I am letting you all know that my poor mother has left this earth; she passed away peacefully surrounded by her family [rephrased]” and “I miss my husband every day, he is getting further away from us...This disease has taken away his mind, I feel exhausted, and life is not fun at all [rephrased].” These topics are related to sentimental changes and offer insights into the deeply personal and heartfelt experiences of community members. Additionally, we identified topics that convey specific emotional actions. For example, topic #2 contains the keywords “cries” and “laughter,” reflecting the diverse sentimental landscape caregivers are facing. A typical initial post from this topic is “When I was a little boy, I often dropped my spoon, and now my old man does the same thing, which makes me laugh and cry at the same time [rephrased].”

In addition to sentiment-related topics, we identified other commonly discussed topics. For example, topic #4 (dementia, advic, diagnos) delved into information related to diagnosis and treatment, providing a relatively objective description. Topic #14 (bed, sleep, shower) centered around caring for people living with ADRD’s daily life, while topic #16 (pay, money, account) was about financial matters. Notably, topic #1 (mother, father, wife) and topic #09 (husband, son, daughter) specifically addressed personal relationships such as those with spouses or adult children. Those topics align with previous studies, which have shown that spousal caregivers and adult-child caregivers make up a significant portion of informal ADRD caregivers [30].

**Figure 4:**
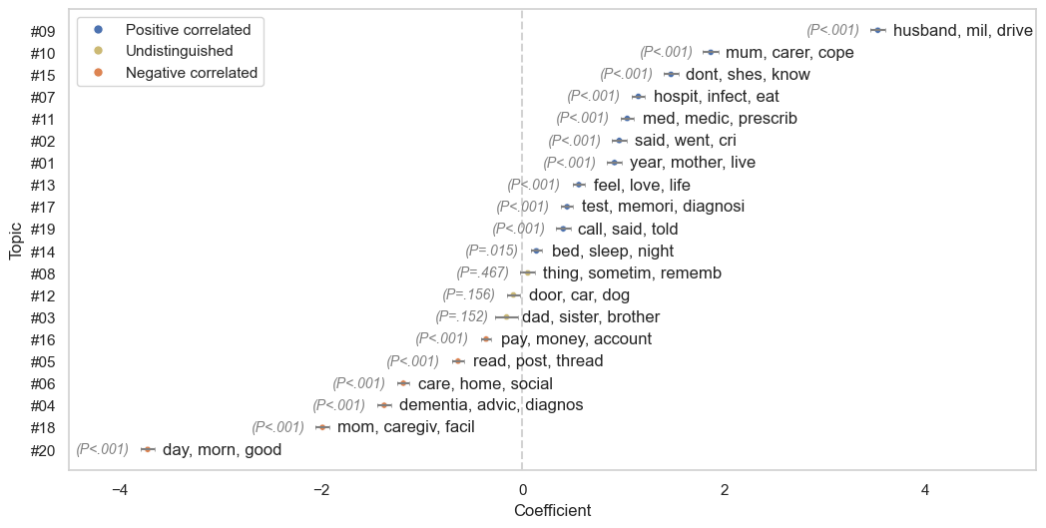
The coefficients of each topic with VADER compound sentiment changes: blue (positive) and orange (negative) dots indicate correlation, while yellow dots indicate undistinguished features where *P*>.05. VADER: Valence Aware Dictionary for Sentiment Reasoning.

To clarify the relationship between the content of initial posts and subsequent changes in sentiment scores, as outlined in RQ2, we present in Figure 4 the influence of each topic on the VADER compound sentiment scores. Blue (positive) and orange (negative) dots represent correlation, each topic’s *P* value is displayed beside its associated keywords. This analysis indicates that most topics are statistically significant (*P*<.001), indicating that various topics are significantly linked to sentiment changes within the topic threads.

When considering specific topics, topic #9 (husband, mil, drive), exhibits the highest positive correlation with changes in the VADER compound score. This topic talks about family relationships and daily life. This suggests that many users of web-based platforms find comfort in sharing their daily experiences and connecting with others. As a result, emotions tend to become more positive as individuals engage in such sharing and communication. By contrast, the most negatively related topic is topic #20 (day, morn, good), which is primarily related to time. This topic illustrates how, as time progresses, the sentiments expressed by caregivers tend to become more negative, possibly due to the progressive nature of dementia. For example, a representative initial post on this topic starts optimistically, *“*Good morning, we are already halfway through March...enjoy the day, it’s looking good [rephrased; VADER compound of 0.97].”

However, as the thread continues, the same author later expresses increasing despair: “Hi [name], not a very good afternoon... Every year I try to finish it by February, but time flies so fast... Things are getting worse for [name]. I hope there is no reincarnation because things are going to be terrible here in a hundred years [rephrased; VADER compound –0.96].”

This transition from a positive to a negative tone, marked by the significant shift in VADER scores, reflects the worsening symptoms over time and the understandable decline in caregivers’ moods.

Furthermore, topics directly tied to sentiment, such as topic #2 (said, went, cri) and topic #13 (feel, love, life), exhibit strong positive associations with sentiment changes. In topic #2, discussions often begin with sentiments of sorrow, as seen in posts like, “My mom has not gotten better...I understand why they call it a long goodbye [rephrased]” and typically conclude with acknowledgments of support, “Thank you so much for your support...I know I am not alone. [rephrased].”

Similarly, topic #13 features initial expressions of emotional turmoil, “This is hard to explain but I’ve been having a weird feeling of loss today...[rephrased]” which transitions into expressions of gratitude, *“*This is great advice [name]...thank you for staying with me [rephrased].” This indicates that the forum effectively caters to individuals seeking to express their emotional experiences.

Topic initiators may receive positive emotional support, likely due to the compassionate and empathetic nature of users in web-based communities; while topics closely related to illness and caregiving facility, such as topic #4 (dementia, advic, diagnostics) and topic #18 (mom, caregiv, facil), display a negative emotional correlation, underscoring the stress and difficulties that caregivers encounter.

It is worth noting that the performance of LIWC tone scores closely mirrors that of the VADER compound hence we did not present the figure here.

### RQ3: Temporal Changes in Caregiver Sentiment

Next, we computed the changes in sentiment according to the VADER compound score and LIWC tone over time. We partitioned users into different timespan groups (from 1 week to 10 years) based on their active time and displayed the sentiment trends in each phase.

**Figure 5:**
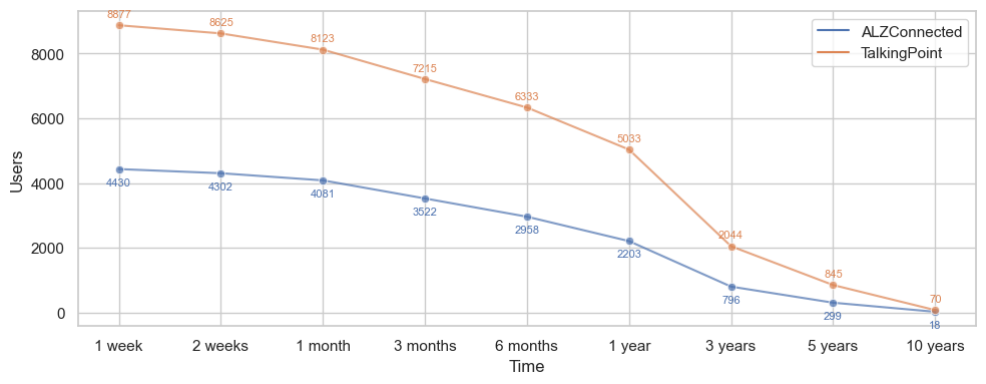
The number of users that are active in each timespan in the 2 web-based communities.

Figure 5 shows the number of users active in each timespan, revealing a notable drop in the number of users active for more than a year. For instance, in the ALZConnected community, out of 4430 users who were active for over a week, only 796 (18%) were active for over 3 years, while only 299 (7%) were active for over 5 years. Interestingly, there is a small fraction of users, 18 (0.4%) out of 4430 users in ALZConnected and 70 (0.8%) out of 8877 in TalkingPoint, who remain active for over 10 years. Due to the substantial reduction in active users after the 1-year mark, our subsequent sentiment trend analysis concentrates on the 1-week to 1-year timespan, capturing a more representative (50%) sample of the community’s active users.

**Figure 6:**
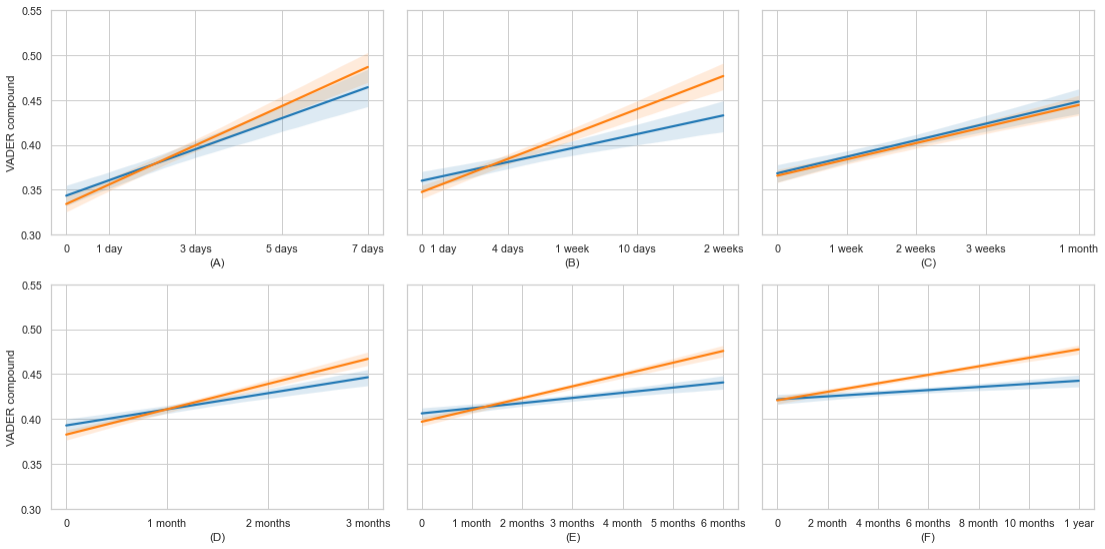
VADER compound sentiment score temporal trend via active time separated into certain time spans. VADER: Valence Aware Dictionary for Sentiment Reasoning.

**Figure 7:**
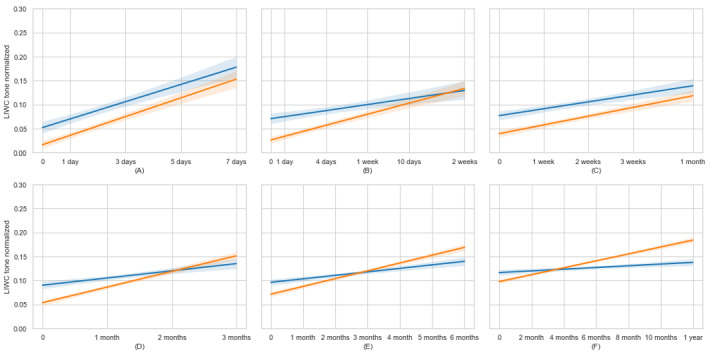
LIWC tone sentiment score temporal trend via active time separated into certain time spans. LIWC: Linguistic Inquiry and Word Count.

This analysis examines the trajectory of sentiment scores among caregivers over time to understand the effect of sustained participation in web-based communities. To improve the readability of the figure, we used linear regression lines, along with linear interpolation and its 95% CI, for the data points in each subplot. Figures 6 and 7 provide a comprehensive view of the VADER compound and LIWC tone sentiment changes, respectively, in the web-based communities. Each subplot demonstrates the trend of sentiment change for eligible users in various timespans.

The analysis reveals an overall positive sentiment trend in both web-based communities, suggesting that engagement in these forums is generally associated with positive emotional expression. Notably, as the active time interval grows, the increase in sentiment weakens. The Spearman rank-order correlation between the VADER compound score and active time within 1 week, 1 month, and 1 year are 0.062, 0.038, and 0.021 (all statistically significant at *P*<.001), respectively. This finding suggests that participation in the web-based community increases a user’s sentiment. However, the effect becomes less pronounced as users spend more time in the web-based community. Third, in both sentiment score measures, there is no substantial difference between ALZConnected and TalkingPoint, showing that both communities provide an environment for ADRD caregivers to express their feelings, supporting the validity of analyzing sentiment changes in ADRD caregivers in either community.

## Discussion

### Principal Findings

This study illustrates patterns related to the sentiment score changes of informal ADRD caregivers within 2 large web-based communities, shedding light on the role of web-based peer support in enhancing their emotional well-being.

Our investigation into the sentiment changes of topic initiators revealed a prompt elevation in sentiment scores compared with their first self-comment. For example, within a TalkingPoint thread spanning 2 years with 8264 comments, the topic initiator initially expressed frustration, stating, “Fed up to the back teeth today. Had a bad night and all day, Mum talking to the clock and asking her Mum to come and get her [VADER –0.36, LIWC –0.22].”

However, after receiving responses from 3 other commenters, one of which is “wish I could draw I had a great image in my head...OK I’ll stop. I just wanted to make you laugh—that’s the best I can come up with [VADER 0.75, LIWC 0.80],” the tone of the conversation shifted positively, as indicated by the first self-comment, “Haha, I feel better now, coming here is like taking a tonic. Thanks, my TP friends. Gotta go, mom has to go to the bathroom so I got the cat out [VADER 0.93, LIWC 0.98].”

This initial boost was followed by a gradual yet continuous improvement as the number of self-comments increased, emphasizing the efficacy of web-based communities in delivering prompt and ongoing peer support to caregivers. Jenkins et al [31] found that web-based support can significantly contribute to well-being, suggesting a similar benefit for ADRD caregivers who actively participate in web-based communities.

Moreover, the continuously increasing trend in sentiment score suggests that web-based peer support is effective in increasing social inclusion [32], which helps to maintain an emotional balance. A plausible explanation is that long-term caregivers in web-based communities who have years of caring experiences, when becoming more capable of caring for people living with ADRD through learning from web-based peers, may sustain stable sentiments. This assumption can be proved by the same example thread as mentioned above, which includes the topic initiator’s 1064 self-comments. As the self-comments progressed from learning new caregiving skills (“Learned a new way to talk to my mom”) to sharing resources (“Same thing happened to me, [name], I recommend you read this book [title], it really helped me”), a strong sense of community and mutual support was ultimately fostered. The last self-comment we collected from a topic initiator was “Hi ladies, hope you all had a great Christmas break, and everyone had a blast. I miss you all and hope the new year is just as great. Big hugs [VADER 0.97, LIWC 0.98].”

As long-term caregivers in web-based communities progressively enhance their caregiving skills through continued engagement with the community [33], their increased proficiency makes their caregiving responsibilities more manageable [34], thus improving their overall quality of life.

However, the sentiment trends of long-term users exhibited a slower sentiment improvement rate compared to short-term users. One possible reason could be that the trajectory of caregiver burdens is highly dynamic and complex due to increased behavioral impairment and decline in functional status in people living with ADRD [35]. This complexity makes it unrealistic to remove all the stressors in this long-term caregiving journal. In other words, informal caregivers will be in stressful situations, and an upper limit of their emotional well-being may exist even when receiving support from other peers in web-based communities. Cultural complexity in caregiving, which includes diverse cultural norms, values, and caregiver expectations, further influences these experiences. It is reflected in the findings of Ajrouch et al [36] that, although often overlooked in research and service delivery, the role of cultural complexity in ADRD care has been recognized.

Furthermore, our topic modeling analysis identified various ADRD caring topics, including those discussing diagnosis, treatment, daily care, and financial matters. We found that topics discussing personal and heartfelt caregiving experiences exhibited a significant positive correlation with sentiment improvement. For example, an initial post in topic #9 aligned with this trend: “My husband has been taking [drug] for anger for about [specific] days now, but it’s not working. Nothing he’s tried seems to work. It drives me crazy [rephrased].” In response, subsequent commentators provided valuable support, sharing experiences with this drug or offering emotional support through sympathy and comfort. These interactions contributed to a more positive sentiment score in the self-comments.

However, our topic analysis also revealed that posts associated with ADRD, and caregiving facilities were correlated with lower sentiment scores, which might be due to the inherent challenges of ADRD caregiving related to these topics. The complexity of ADRD poses significant emotional and psychological challenges for caregivers [37]. For instance, an indicative initial post from topic #4 reads, “Hi, I am a full-time carer for my [age]-year-old husband who has vascular dementia and is profoundly deaf. Is there anyone on the forum who is in a similar situation? Thank you [rephrased].” The responses to this post included, “Hi [topic initiator name], my [age]-year-old husband has vascular dementia but without the added complication of being deaf. He lives so much in his own world most of the time that he often seems to be deaf, though. It doesn’t feel nice [rephrased].” These interactions led to the topic initiator’s self-comment, “Thank you all for your responses. It seems to be totally deaf, and dementia is quite uncommon. You are right—I do often feel lonely and isolated, as he must as well. I feel very sad [rephrased].” This is a typical example of negative sentiment change resulting from communication with other users of web-based platforms in the community. ADRD caregivers often witness their loved ones struggling with a loss of identity and independence. As caregivers provide care and support for individuals with ADRD, they often experience feelings of sadness, frustration, and helplessness [38]. In this situation, the decrease in sentiment may be caused by the continued narrative of their negative caregiving experience. However, sharing these challenging experiences with other web-based peers may foster a sense of belongingness among caregivers [39,40], which may lead to a long-term sentiment improvement, as shown in our sentiment temporal trends analysis.

### Limitations and Future Works

While sentiment analysis tools provide valuable insights, they may not fully capture the intricate nuances and complexity of human emotions within the ADRD caregiving context. Future analyses may consider combining supervised machine learning for more precise sentiment classification. Although our study identified a correlation between topic initiators and positive sentiment change within threads, it is important to delve deeper into understanding whether web-based interactions directly cause sentiment changes. Our future research will expand the analysis to include all comments within a thread, thus offering a more comprehensive view of the community’s support structure. This approach will allow us to better understand the overall sentiment dynamics and support mechanisms across the platform, addressing the skewed perspective that may arise from focusing solely on self-comments.

The use of STM in our analysis, while powerful for identifying dominant themes from large text corpora, can also present challenges. These models may generate overlapping themes that do not distinctly separate different but related caregiving aspects, due to the unsupervised nature of the topic generation process. This overlap can sometimes obscure the clarity of how specific topics impact caregiver sentiment. Future studies might explore refined modeling techniques that can more effectively differentiate closely related topics or apply hierarchical models to capture nested thematic structures.

Also, our study only examined the registered users who actively write posts on web-based platforms. Since both web-based communities are open to anyone, the data primarily reflect the experiences of active contributors, potentially overlooking the perspectives of passive users or those who may face barriers to participation. This selective participation may concentrate the content creation among a small subset of highly active users, which might narrow the findings to this more vocal group. Additionally, the lack of demographic data on participants limits the generalizability of our findings across diverse caregiver populations, which could result in an unrepresentative sample. It will be interesting to investigate how discussions on web-based platforms, as collective knowledge, can influence the emotional well-being of all caregivers, including those who only observe interactions without contributing directly. Such findings will help to expand the impact of web-based peer support, which is unique to open web-based communities.

### Conclusions

To the best of our knowledge, this is the first study to investigate how sentiment changes among informal ADRD caregivers within 2 open, large, existing web-based communities using computational methods. We observed improved sentiment score trends at both the topic thread and community levels, highlighting the positive impact of web-based peer support for both short-term and long-term caregivers on web-based communities. However, we did find some topics that are negatively associated with sentiment improvement, which reflects the complexity of some caregiving burdens that might not be easily solved at the emotional level. Overall, our findings indicate that peer support in web-based communities can be powerful in assisting informal ADRD caregivers.

## Abbreviations

AD: Alzheimer disease
ADRD: Alzheimer disease and related dementias
ETP: expected topic proportions
LIWC: Linguistic Inquiry and word count
RQ: research question
STM: structural topic modeling
VADER: Valence Aware Dictionary for Sentiment Reasoning
